# A clue to the triples from an echo

**DOI:** 10.1002/joa3.12802

**Published:** 2022-12-20

**Authors:** Mathurin Suwanwalaikorn, Narut Prasitlumkum, Ronpichai Chokesuwattanaskul

**Affiliations:** ^1^ Faculty of Medicine Chulalongkorn University Bangkok Thailand; ^2^ Department of Cardiology University of California Riverside Riverside California USA; ^3^ Division of Cardiology, Department of Medicine, Faculty of Medicine Chulalongkorn University and King Chulalongkorn Memorial Hospital Bangkok Thailand

**Keywords:** AV nodal physiology, ECG, electrocardiogram, left septal fascicular block, ventricular echo beat

A 60‐year‐old female patient with no significant past medical history presented to the hospital for an annual health checkup. A physical examination and laboratory panels were within normal limits. Nonetheless, an abnormal 12‐lead electrocardiogram (ECG) was noted and, hence, a request for cardiology consultation was made to evaluate the findings. What is your diagnosis? What is the implication?

Overall, the ECG was consistent with sinus rhythms with premature ventricular contraction (PVC) bigeminies. At first glance, it can be mistakenly interpreted as a PVC interpolation after bigeminies owing to its similarity (Figure 1). Considering post‐PVC RR intervals (R5–R6 and R10–R11), it appeared implicitly longer than those pseudo‐interpolated PVC beats (R3–R5 and R8–R10). Further, each coupling interval was varying, as illustrated in Figure 2A. From these rationales, interpolated PVC deems unlikely. In fact, these were groups of retrograde P waves that antegradely conduct through the atrioventricular (AV) node producing an echo beat, as illustrated in Figure 1 and its description. With predominant negative axis of the P wave (lead II, III, and aVF) and equal coupling intervals between the PVCs and the retrograde P within each set of the extrastimuli, these two findings together strongly convinced that the electrical impulses were most likely carried retrogradely from the ventricle to the atrium and favored retrograde P than supraventricular extrastimuli. This ECG pattern has been iterated as one of the manifestations of dual AV nodal pathways physiology.^1^ At least two different intra‐AV nodal pathways may be discovered should an electrophysiology (EP) study be performed.

**FIGURE 1:**
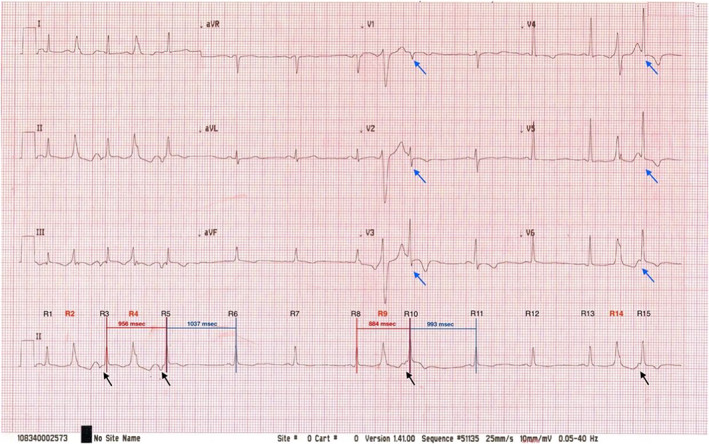
12‐Lead electrocardiogram recorded during a health checkup. Lead II: four PVCs were found (labeled in red) followed by retrograde P waves (black arrow) and ventricular echo beats, respectively. Precordial leads: Left septal fascicular block pattern manifested as loss of septal q wave in lead V5–V6, tall R in lead V2, and progressive increase of R voltage from V1 to V3 and decreasing from V5 to V6 (blue arrows)

**FIGURE 2:**
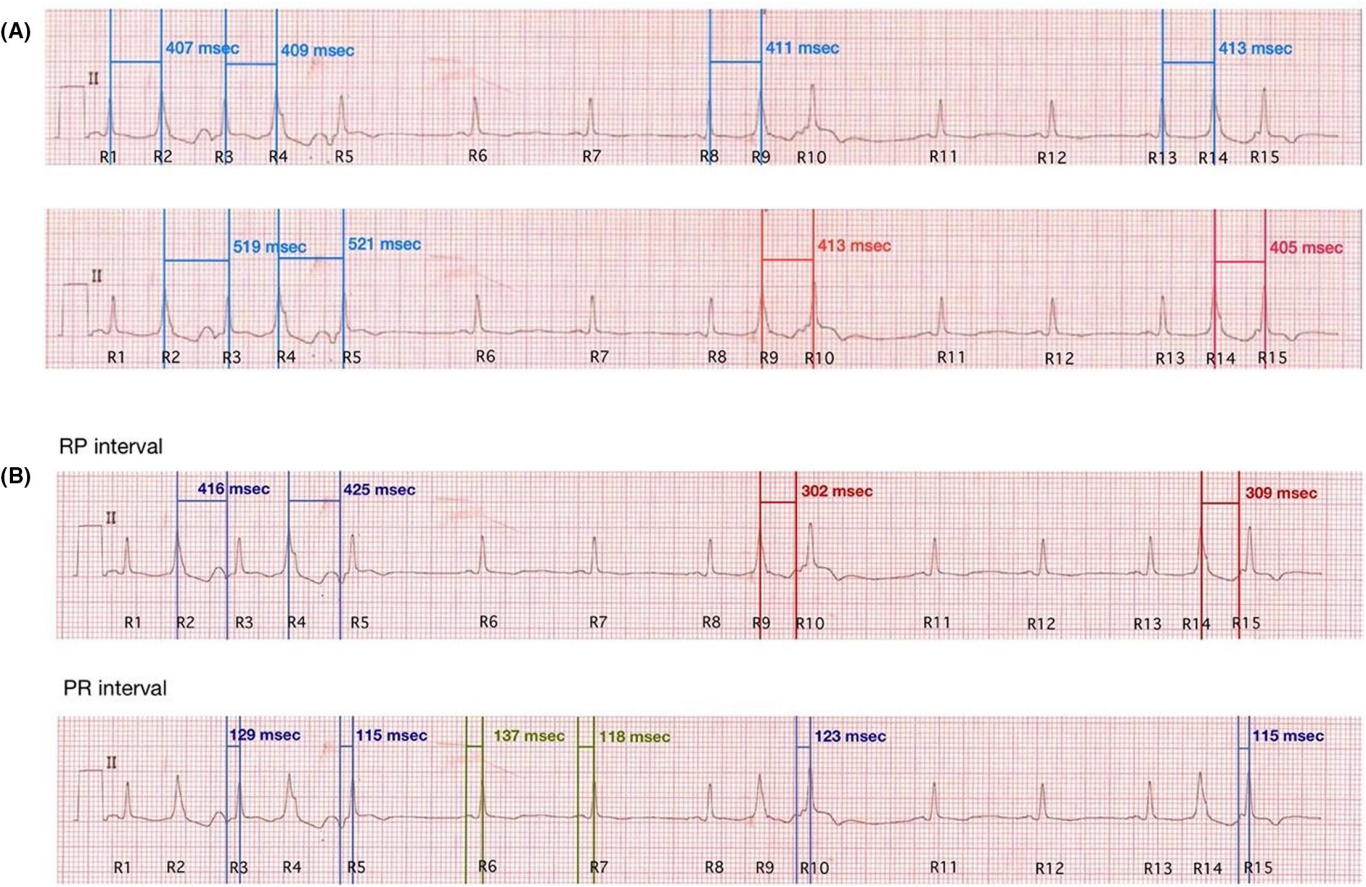
(A) Measures of coupling interval. Top tracing: fixed coupling PVC at 407–413 ms. Bottom tracing: two sets of coupling intervals of beats after PVC at 519–521 ms and 405–413 ms(B) RP and PR segments duration. Top tracing: variable R–P intervals occurred in two distinct intervals (416–425 ms and 302–309 ms). Bottom tracing: constant P–R intervals for all four groups (labeled in blue), which were approximately the same as P–R intervals of the remaining sinus beats, varying from 118 to 137 ms (labeled in green)

Considering the retrograde P waves preceding R3, R5 and R10, R15, there were two main reasons that could explain the differences in their morphologies. First, the superimposition of the preceding T waves and their aftercoming P waves may render their appearances. Second, the filtering effects from the ECG device may affect the P wave configurations.^2^ Imperfectly, the denoising process itself may derange P‐QRS contours, as portrayed in our report. Therefore, P waves following R3, R5 and R10, R15 were most likely retrograde P waves.

Usually, an electrical impulse of PVC passes through the AV node retrogradely and later may excite the atrium muscle, resulting in a retrograde P wave. On the other hand, interpolated PVC may ensue if the retrograde conduction falls within the AV node refractory period. On a closer look, there were two different coupling intervals of PVCs following the narrow QRS complex: 520 and 410 milliseconds (Figure 2A). We segregated the coupling intervals of these beats C into RP and PR segments and measured their durations (Figure 2B). Following this maneuver, V‐A conduction times were noted at two distinct intervals (416–425 ms and 302–309 ms) implying the presence of multiple retrograde pathways.^3^ For this reason, it is reasonable to presume that this patient has two different retrograde AV nodal pathways. Of note, P–R intervals were measured, invariably ranging around 115–129 ms and 115–123 ms. These durations were approximately the same as the P–R interval of the following sinus beats (118–137 ms), which implies that there is a sole antegrade AV conduction pathway.

Furthermore, the vignette ECG entailed the decremental conduction property of the AV nodal, best demonstrated in Figure 2. When measuring the durations between R2‐R3, R4‐R5, and the PR intervals of R3 and R5, R2–R3 (416 ms) was shorter than R4–R5 (425 ms) but the PR interval of R3 (129 ms) was comparatively longer than the PR interval of R5 (116 ms). Given the slight differences in these durations, the possibility of measurement error cannot be excluded. Knowing this limitation, several measurements by our institution experts were made yielding the exact numbers.

Figure 3 illustrates a simple hypothetical model of intra‐AV nodal physiology. At each time, a PVC creates an electrical impulse that passes through the AV node. One of the two selected retrograde pathways (*α* or *β*) must be in the refractory period. Thus, the returning conduction through that pathway is not possible and the impulse must then transmit through a different pathway. As we can observe only one set of P–R intervals, we can conjecture pathway *γ* is the shared pathway that receives impulses transmitted from either *α* or *β* and antegradely conducts through the AV node, forming the reciprocal beats.

**FIGURE 3:**
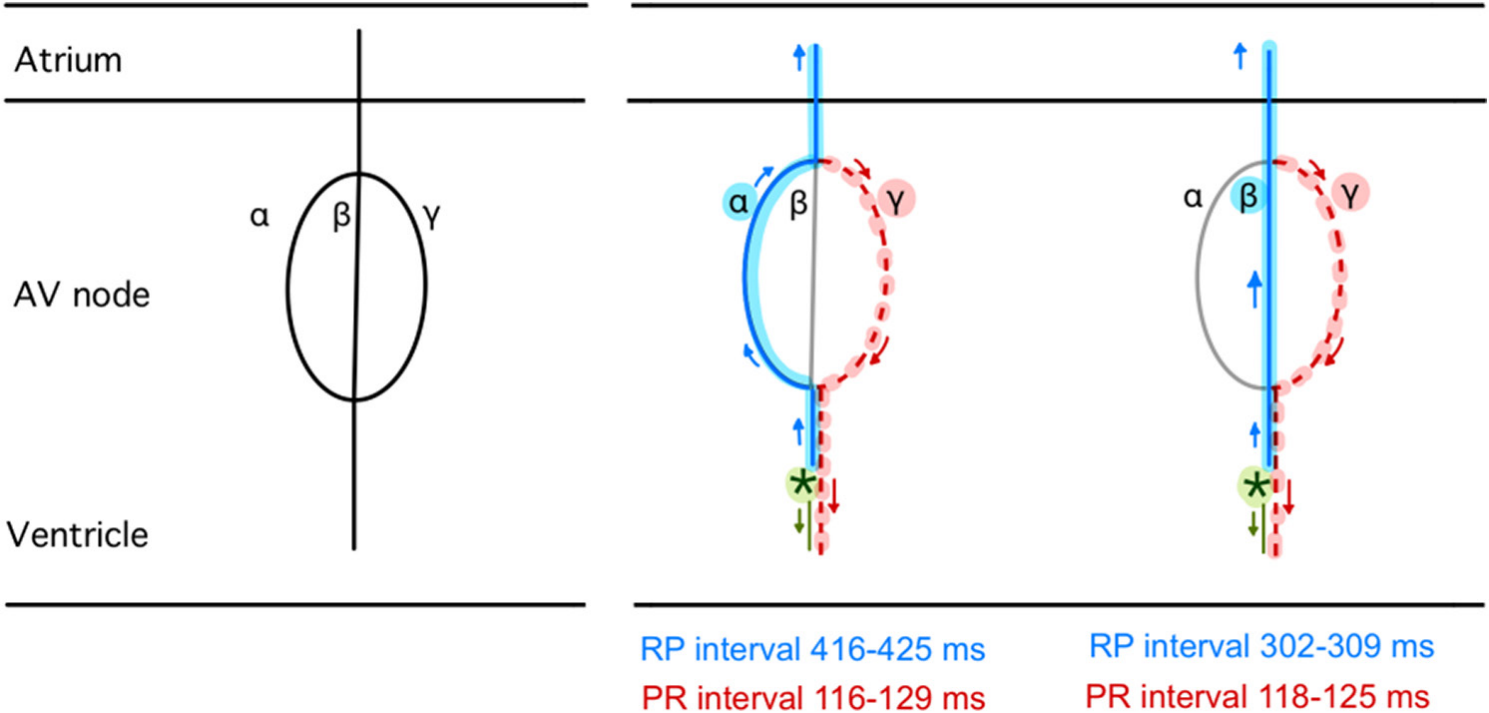
Hypothetical model of triple AV nodal pathways; three pathways of conduction are assumed (*α*, *β*, *γ*). Asterisk (*): ventricular ectopic foci. Solid blue line: retrograde conduction. Red dash line: antegrade conduction

In addition to the intriguing multiple AV nodal pathways, this ECG demonstrated the aberrant conduction of antegrade conducted ventricular echo beats, best depicted in the precordial leads (Figure 1). With the loss of septal q wave in left precordial leads (V5, V6, and I), and a prominent tall R in lead V2. These findings suggested that there was anteriorization of the QRS forces in the horizontal plane, which possibly occurred as a result of delayed activation in the left anteroseptal area.^4^, ^5^ Hence, we suspected that the aberrant conduction of antegrade conducted beats was probably due to a functional block of the left septal fascicle.

To prove our postulation, an EP study is required to determine the nature of the triple AV nodal physiology. At this stage, we contemplated that this is the highest likely diagnosis based on the existing evidence and logical explanations. Undeniably, other possibilities cannot be completely excluded despite less likelihoodness. These included premature atrial contraction with aberrancy and ectopic junctional rhythm with aberrancy. As the patient was asymptomatic and only referred for abnormal ECG evaluation, this did not meet any clinical indications for an EP study, and we decided not to offer the procedure on the initial visit. Instead, we recommended a follow‐up with our clinic and would only consider an EP study, should there be an apparent indication.

This case report proposed the possibility of not just dual, but triple AV nodal physiology. Several ECG findings, in this case, satisfy our postulation, denoting the existence of multiple AV nodal conduction pathways.

## AUTHOR CONTRIBUTIONS

Mathurin Suwanwalaikorn wrote the paper. The remaining authors contributed to the scientific revision of this case.

## CONFLICT OF INTEREST

The authors declare that they have no conflict of interest to disclose.
